# Beyond Moco Biosynthesis―Moonlighting Roles of MoaE and MOCS2

**DOI:** 10.3390/molecules27123733

**Published:** 2022-06-10

**Authors:** Tamaki Suganuma

**Affiliations:** Stowers Institute for Medical Research, 1000 E. 50th Street, Kansas City, MO 64110, USA; tas@stowers.org

**Keywords:** MoaE, MOCS2, MPTAC, ATAC, sulfur catabolism, nucleotide metabolism, neurodegenerative disease

## Abstract

Molybdenum cofactor (Moco) biosynthesis requires iron, copper, and ATP. The Moco-containing enzyme sulfite oxidase catalyzes terminal oxidation in oxidative cysteine catabolism, and another Moco-containing enzyme, xanthine dehydrogenase, functions in purine catabolism. Thus, molybdenum enzymes participate in metabolic pathways that are essential for cellular detoxication and energy dynamics. Studies of the Moco biosynthetic enzymes MoaE (in the Ada2a-containing (ATAC) histone acetyltransferase complex) and MOCS2 have revealed that Moco biosynthesis and molybdenum enzymes align to regulate signaling and metabolism via control of transcription and translation. Disruption of these functions is involved in the onset of dementia and neurodegenerative disease. This review provides an overview of the roles of MoaE and MOCS2 in normal cellular processes and neurodegenerative disease, as well as directions for future research.

## 1. Introduction

The synthesis of protein, DNA, and RNA is the foremost consumer of the cellular ATP supply [1]. Chromatin processes, including nucleosome remodeling, ATP-dependent nucleosome assembly, histone phosphorylation and ubiquitination, and transcription, also greatly utilize ATP. Therefore, chromatin status reflects the cellular energy status and participates in energy dynamics. The repeating structural unit of chromatin is the nucleosome, which consists of a histone octamer composed of two copies each of the core histones H2A, H2B, H3, and H4, wrapped with 147 bp of DNA [2,3]. Posttranslational modifications of the N-terminal extensions of the core histones (the histone tails) modify the stability of the nucleosome and the accessibility of transcription factors and chromatin modifiers to their binding sites [1]. In addition, DNA modifications such as methylation of cytosine, oxidation of methylated cytosine, and methylation of adenine to N^6^-methyladenine alter chromatin structure and gene expression [4]. Thus, chromatin modification pivotally regulates transcription, gene expression, and epigenetics.

Metabolites are essential cofactors for histone and nucleosome modifications. Such metabolites include acetyl coenzyme A (acetyl-CoA) for histone acetyltransferases (HATs), which transfer an acetyl group to an epsilon amino group of a lysine side chain in the histone tails, and S-adenosyl methionine (SAMe) for histone methyltransferases (HMTs), which transfer a methyl group to amino groups in lysine or arginine residues in the histone tails. These reactions are reversible via histone deacetylases (HDACs) [5] and histone demethylases, respectively [1,5]. Therefore, chromatin modifications and transcription respond to cellular metabolism. Accumulation of cytoplasmic acetyl-CoA promotes transportation of acetyl-CoA into the nucleus, leading to nuclear histone acetylation, which activates transcription [6]. When cells are starved, acetyl-CoA from fatty acids becomes a source of ATP generation; therefore, nuclear acetyl-CoA levels are reduced, resulting in repression of gene expression [6].

Many enzymes regulating chromatin modification respond to cellular signals by forming protein complexes [1,7]. Forming chromatin-modifying complexes improves the catalytic activities of these enzymes and enables crosstalk between chromatin modifications [8]. In *S. cerevisiae*, the serine-responsive SAM-containing metabolic enzyme (SESAME) complex, which contains serine metabolic enzymes, SAMe synthetases, an acetyl-CoA synthase, and a pyruvate kinase, interacts with the Set1 histone H3K4 methyltransferase complex, which requires SAMe synthesized from SESAME [9]. The pyruvate kinase (Pyk1) in SESAME phosphorylates histone H3T11 by utilizing phosphoenolpyruvate (PEP) [9]. The interaction of SESAME with Set1 regulates crosstalk between H3K4 methylation and H3T11 phosphorylation by sensing glycolysis and glucose-derived serine metabolism [9]. Notably, chromatin modifiers also contain nucleotide biosynthetic enzymes, as seen in the heteromeric complex of 5′-monophosphate synthase (GMPS) with ubiquitin protease 7 (USP7) in *Drosophila* and in mammals [5].

The Moco biosynthetic enzyme MoaE was discovered as a subunit of the Ada2a-containing (ATAC) histone acetyltransferase complex in *Drosophila* [10]. Forming MPT synthase, a heterodimer of MoaD and MoaE, is essential for Moco biosynthesis [11]. Human MPT synthase consists of a heterodimer of MOCS2A and MOCS2B, which are both encoded by one gene, *MOCS2* [12]. Recent studies have revealed the functions of ATAC in parallel with the unexpected phylogeny of MoaE and its roles in signaling, metabolism, and translation. Further studies on the crosstalk of the protein complexes uncovered the functions of MoaE in specific cellular signaling in higher eukaryotes. In this review, we summarize the functions of MoaE and MOCS2 beyond Moco biosynthesis in *Drosophila* and in humans and discuss open questions and future directions for the understanding of roles of Moco biosynthetic enzymes and molybdenum enzymes in cellular processes.

## 2. The Discovery of MoaE in a Histone Acetyltransferase Complex in *Drosophila*

**Figure 1:**
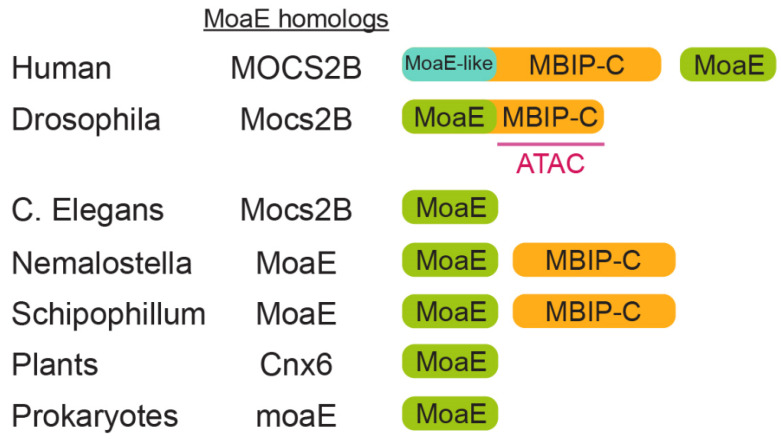
MBIP may be evolutionarily derived from MoaE. Schematic representation of phylogenetics of the MoaE and MBIP domains. The MBIP sequence in *Drosophila* Mocs2B/dMoaE, which is conserved in the C-terminus of human MBIP, is essential for incorporation into ATAC (red line).

Chromatin-modifying enzymes function within multi-subunit complexes that are conserved from yeast to humans, as discovered through biochemical purification of these enzymes. The Gcn5 histone acetyltransferase was found to be a component of the Spt-Ada-Gcn5-acetyltransferase (SAGA), SAGA-like (SLIK), and transcription ADAptor (ADA) complexes in yeast and a component of the SAGA and ATAC complexes in flies and humans [10,13,14,15,16,17]. The ATAC complex was discovered in *Drosophila melanogaster* and is an essential HAT complex with a size of 1.3 MDa, and it contains 13 subunits, including two HATs, Gcn5, and Atac2/KAT14 [10]. While Gcn5 acetylates H3K9 and H3K14 in *S. cerevisiae* and *Drosophila,* as well as in humans, Atacs2 is a higher-eukaryote-specific HAT [10]. Recombinant Atac2 preferentially acetylates histone H4, and mutation of Atac2 abrogates H4K16 acetylation in *Drosophila* embryos [10]. Thus, ATAC acetylates both H3 and H4. H4K16 acetylation is a key player in dosage competition; however, whether ATAC is involved in dosage competition is unknown. ATAC does not show nucleosome-remodeling activity itself, but facilitates nucleosome sliding by the ISWI, SWI–SNF, and RSC complexes [10]. Interestingly, ATAC contains Mocs2B/dMoaE in *Drosophila* [10]. ATAC was then found in humans [17,18], and human ATAC (hATAC) was found to consist of 14–15 subunits, including two HATs, ATAC2/CRSP2-BP, and GCN5/PCAF. However, hATAC contains the mitogen-activated protein kinase (MAPK)-upstream protein kinase-binding inhibitory protein (MBIP) instead of MOCS2B (the human homologue of Mocs2B/dMoaE, as described below) [17,18]). Indeed, the conserved MBIP sequence in *Drosophila* Mocs2B/dMoaE—but not the MoaE domain—is essential for incorporation into ATAC [19] (Figure 1). These observations raised the following question: What role does MoaE play in the functions of ATAC?

## 3. Evolution of MoaE and MBIP

*Drosophila* melanogaster MoaE/Mocs2B/CG10238 (dMoaE) consists of two domains: the MoaE domain in the N-terminal region and the MBIP domain in the C-terminus (Figure 1). The homodimerization of purified recombinant Mocs2B/dMoaE and the direct interaction of Mocs2B/dMoaE with Mocs2A/dMoaD suggest that Mocs2B/dMoaE resembles prokaryote MPT synthase, as shown in its X-ray crystal structure as a heterotetrameric protein in which the C-terminus of each MoaD subunit is inserted into MoaE to form the active site [20,21]. The MBIP sequence in Mocs2B/dMoaE is homologous to human MBIP (Figure 1). MBIP is conserved in many Metazoa, but has been thought to lack homology in any other proteins [21]. MoaE homolog and MBIP are separate proteins in humans, but are a single fused protein in *Drosophila*. Interestingly, the vertebrate N-terminal regions of MBIP proteins are distantly related to MoaE [21] (“MoaE-like”, as shown in Figure 1). Seven residues implicated in molybdopterin (MPT) binding and catalysis in MoaE are conserved in Mocs2B/dMoaE; however, only three such residues remain in other MBIP proteins (MBIP is found in most animals) [21]. Seven of the ten residues mediating the MoaE–MoaD interaction in bacteria are conserved in the N-terminal regions of MoaE-like moieties of MBIP proteins [21]. Therefore, N-terminal regions of MoaE-like moieties of MBIP proteins likely lost catalytic activity as MoaE, but may enable binding to MoaD. The conclusion drawn is that MBIP is evolutionally derived from ancient MoaE, and that standalone MBIP lost catalytic activity as MoaE. While these features of MBIP were becoming clear, the following question arose: Is MoaE is involved in the functions of MBIP?

## 4. Roles of MoaE and MOCS2 in Signaling in *Drosophila* and Humans

Cells exposed to increases in extracellular osmolality respond through rapid activation of stress-activated MAPKs (SAPKs), including c-Jun-NH2-terminal kinase (JNK) and p38 MAPKs [22,23]. Osmotic stress activates JNK by phosphorylation, which, in turn, phosphorylates c-Jun transcription factor and facilitates its transcriptional activity [24,25]. Several MAPKs, including ERK1/2, p38, and JNK, bind to and regulate transcription of the insulin gene [26]. Hence, MAPKs are key players in regulating gene expression in response to cellular signaling. In *Drosophila*, ATAC is required for Jra/c-Jun occupancy of osmotic-stress-responsive genes and for H4K16 acetylation at the *Jra*/*c-Jun* enhancer, promoter, and transcribed sequences [19]. However, rapid stress response upon osmotic stress is also inhibited by ATAC. Under conditions of osmotic stress, ATAC colocalizes with Jra/c-Jun, recruits upstream kinases, including JNK, and suppresses further activation of JNK [19]. Remarkably, an ATAC subunit, Mocs2B/dMoaE, plays a role in suppressing the phosphorylation and activation of JNK [19]. This JNK-suppressive activity is conserved in hATAC [19].

MBIP was initially identified as a MAPK upstream kinase (MUK) binding partner in a yeast two-hybrid screen [27]. Notably, the MBIP sequence in Mocs2B/dMoaE is required for incorporation into ATAC in *Drosophila* [19]. Consistently, hATAC contains MBIP, but not MOCS2B. However, the MoaE domain in Mocs2B/dMoaE catalyzes the inhibition of JNK phosphorylation, suggesting that MoaE compensated for the roles of MBIP before the evolutionary appearance of standalone MBIP. Overall, Mocs2B/dMoaE and MOCS2 play an important role in chromatin regulation and gene expression via its interaction with ATAC.

These discoveries demonstrated that ATAC functions as a positive transcription coactivator of JNK target genes and provides negative feedback for inhibition of the JNK signaling pathway. The JNK pathway and c-Jun have been shown to suppress apoptosis and cellular senescence in a p53-dependent manner [28,29,30]. Importantly, knockdown of ATAC subunits leads to a decrease in p53 expression due to increased JNK activity [19], suggesting that ATAC promotes p53-dependent cellular senescence in response to stress.

Cellular senescence has opposing effects: While acute senescence contributes to tissue remodeling during wound healing and an anti-tumor microenvironment by altering macrophage polarization [30,31], lingering senescence contributes to chronic inflammation, referred to as “inflammaging” [31]. Interestingly, dextromethorphan (DM), which was discovered as a senolytic compound that reduces senescent cells, has been used to treat Moco deficiency (MoCD) [31,32]. DM has also been used to treat symptoms associated with neurotoxicity in Parkinson’s disease and autism [33]. DM is a non-opioid antitussive agent [33,34] and a noncompetitive receptor antagonist of N-methyl-D-aspartate (NMDA), and it is applied in combination with quinidine, a cytochrome P450 2D5 inhibitor. It remains unknown whether DM merely removes the cells triggering inflammaging. Macrophages in the brain (microglia) contribute to removing debris from the brain, and accumulation of such debris is associated with Alzheimer’s disease (AD). Macrophage polarization is regulated during chromatin remodeling by SWI/SNF [35,36]. One possibility is that ATAC contributes to the chromatin remodeling for macrophage polarization and suppresses JNK signaling to promote acute senescence. In this case, ATAC may help lessen the neurotoxicity observed in MoCD. A study addressing whether DM enhances/enables the activity of ATAC is needed.

## 5. MPT Synthase and ATAC Coordinate Transcription and Translation in Humans

**Figure 2:**
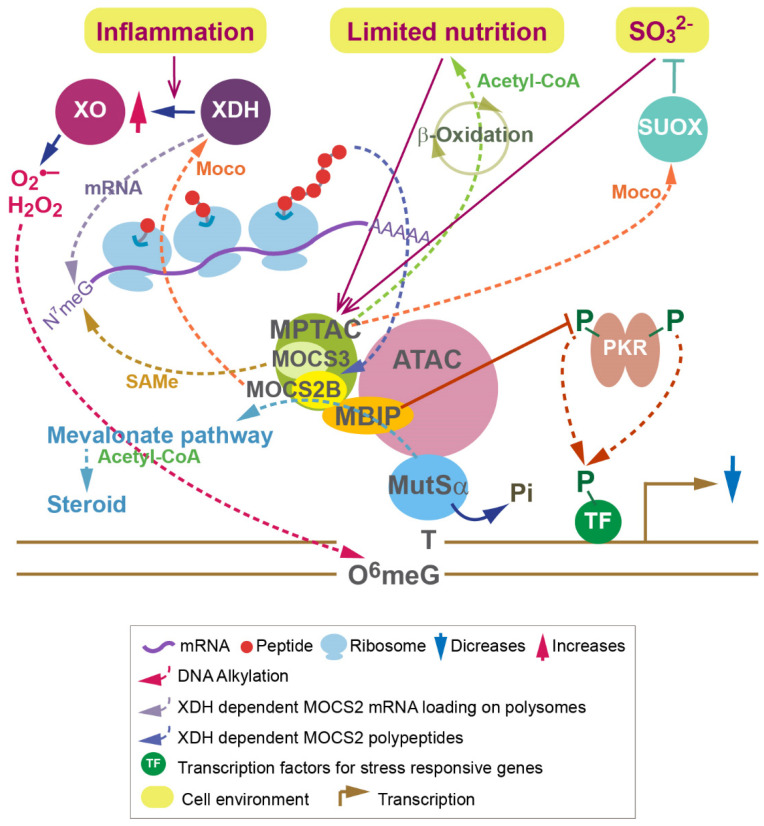
MOCS2 translationally responds to inflammation, DNA alkylation signaling, and nutritionally challenging environments (e.g., limited acetyl-CoA and sulfur amino acids). MPTAC regulates sulfur amino acid catabolism in which SUOX catalyzes the oxidation of sulfite, which contains a sulfite ion (SO_3_^2−^), to sulfate (SO_4_^2−^) in the terminal step. This prevents accumulation of toxic sulfite and ROS generated from disordered sulfur amino acid catabolism. XDH activity requires Moco. XDH is converted into XO in the inflammatory environment. Increased XO activity produces superoxide ion (O_2_^•–^) and hydrogen peroxide (H_2_O_2_), sources of alkylating agents that trigger DNA alkylation damage. MutSa responds to DNA alkylation damage (O^6^-methylguanine, O^6^meG) in a MPTAC- and ATAC-dependent manner. DNA alkylation damage signaling by the association of MutSa, MPTAC, and ATAC promotes mevalonate pathways, leading to sterol synthesis and resulting in an anti-inflammation response and a reduction in alkylating agents. XDH loads MOCS2 mRNA onto polysomes, promoting MOCS2 protein synthesis. Therefore, XDH promotes DNA alkylation damage signaling via promotion of MPTAC expression, which is essential for MutSa stability. Overall, in inflammatory environments, XDH is converted into XO, which induces apoptosis of toxic cells carrying mutations via the production of alkylating agents. MPTAC promotes fatty acid β-oxidation, which generates acetyl-CoA in nutritionally challenging environments. Acetyl-CoA is essential for initiation of the mevalonate pathway. MPTAC also maintains cellular levels of SAMe, which is required for methylation at N^7^G in mRNA. Thus, MOCS2 in MPTAC coordinates the utilization of acetyl-CoA and SAMe.

JNK is an inflammatory kinase that regulates insulin and metabolism [37]. dsRNA-dependent protein kinase (PKR) phosphorylates JNK and eIF2α in response to nutrient and inflammatory stress [38]. PKR was initially found to recognize pathogens and to regulate the immune response against viral infections [39]. GTP-bound eIF2α assembles with the methionyl initiator tRNA to form a translation-initiation ternary complex (TC), which promotes translation initiation [40]. PKR and other stress-activated kinases phosphorylate eIF2α, resulting in sequestration of the nucleotide exchange factor eIF2B, leading to attenuation of translation [41]. Interestingly, MBIP interacts directly with MOCS2B and PKR, suppresses latent PKR autophosphorylation, and prevents its downstream phosphorylation of JNK and eIF2α [42] (Figure 2). The association of MPT synthase with ATAC and the subsequent inhibition of PKR promote the translation initiation of iron-responsive mRNA [42]. Similarly, a recent study showed that MOCS2 is critical for mRNA translation [43]. Thus, MPT synthase and ATAC regulate latent PKR signaling and coordinate transcription and translation initiation [42] (Figure 2).

Sulfur is transferred to the nucleotide in tRNA through a protein-ubiquitylation-like modification called urmylation. Two sets of thiouridines have been found at the wobble bases of tRNAs: 5-methoxycarbonylmethyl-2-thiouridine (mcm5s2U34) in cytoplasmic tRNAs and 5-carboxymethyl-2-thiouridine (cmnm5s2U34) in mitochondrial tRNAs [44,45]. MOCS3 interacts with URM1 in humans [46,47]. Remarkably, MOCS3 adenylates both MOCS2A and URM1, leading to a sulfur transfer for formation of a C-terminal thiocarboxylate group in each protein [48]. The regeneration of the thiocarboxylate group on MOCS2A after releasing MPT may require MOCS3 based on studies in *E. Coli* [49,50]. Therefore, the rate of Moco biosynthesis may influence 2-thiouridine formation, although MOCS3 appears to have two different functions. In *S. cerevisiae*, thiolation of wobble-uridine (U34) nucleotides present on lysine, glutamine, or glutamate tRNAs is important for efficient translation of genes enriched in codons of these amino acids [51]. In the thiolation reaction, Urm1p forms a thioester with the E1-like protein Uba4p [52]. Importantly, the thiolation status of these U34 nucleotides, as well as Uba4p protein levels, is downregulated upon methionine and cysteine starvation to reduce sulfur consumption and cell growth [51]. However, reduction of these thiolations leads to compensatory increases in the expression of enzymes involved in methionine, cysteine, and lysine biosynthesis. Therefore, tRNA thiolation mediates cell growth modulation by sensing sulfur amino acids levels [51]. Similar biological roles of tRNA thiolation are expected in mammals despite Moco biosynthesis not being conserved in *S. cerevisiae* [11]. Since MPT synthase responds to sulfur amino acid catabolism (described in the next section) and links transcription to translation initiation, it would be interesting to know whether the interaction of MOCS3, MPT synthase, and ATAC modulates tRNA thiolation levels in nutritionally challenging environments.

## 6. Functions of MPTAC in Metabolism Are Disrupted in Dementia

The regulation of PKR through the association of MPT synthase and ATAC suggested that MPT synthase may sense metabolism. MPT synthase is found in a 274 kDa cytoplasmic complex called the MPT synthase associating complex (MPTAC), which contains MOCS3, DBN1, CLNS1A, SNRPD2, SNRPB/B’, and HSD17B10 [53]. Due to the observations of increases in the levels of cysteine and homocysteine and decreases in the expression levels of sulfite oxidase (SUOX) in MOCS2 knockdown cells, MPTAC was found to regulate sulfur amino acid catabolism, in which SUOX catalyzes the terminal step, preventing accumulation of sulfite (SO_3_^2−^) and reactive oxygen species (ROS) [53] (Figure 2). Interestingly, MPTAC is crucial for regulating cellular S-adenosylmethionine (SAMe) levels [53]. MPTAC-dependent SAMe is required for the arginine methylations of SNRP splicing factors in pre-mRNA splicing, including amyloid precursor protein (APP) pre-mRNA splicing [54]. MOCS2-knockdown leads to the creation of abnormal APP proteolytic fragments that are observed in the platelets of AD patients [53,55]. Amyloid beta (Aβ), which is generated by the sequential cleavage of APP and is found in the brains of AD patients, accumulates in MOCS2-knockdown cells [53]. Expression of the MPTAC subunit, DBN1, has been found to be decreased upon Aβ deposition in hippocampal neurons [56]. DBN1 expression levels were also reduced in MOCS2-knockdown cells. Hence, MPTAC prevents events that occur in the onset of AD [53].

A global metabolomics analysis in MOCS2-knockdown cells suggested the crucial roles of MPTAC in fatty acid β-oxidation upon glucose and pyruvate depletion [53] (Figure 2). Moreover, MOCS2 is essential for the pathways depending on acetyl-CoA production from the TCA cycle, and MOCS2-knockdown elevates urea cycle activity. Hence, prevention of ROS production by MPTAC may be critical when cells are under conditions requiring recycled acetyl-CoA (Figure 2).

An additional role of MPT synthase in mRNA translation is that SAMe is used for mRNA synthesis in an MOCS2-dependent manner (Figure 2). The 5′ cap in mRNA consists of N^7^-methylguanine (N^7^meG) connected by a 5′-to-5′ triphosphate bridge to the first mRNA nucleotide [57]. The N^7^meG cap prevents mRNA degradation [57]. MOCS2-dependent SAMe is essential for the methylation of N^7^G [43]. Hence, MOCS2 is also required for mRNA processing. Subsequently, MOCS2 contributes to the synthesis of polyamines, which influences nucleolar RNA production [43]. Polyamine transfer is also connected with the purine salvage pathway [43]. MOCS2 is essential for cellular guanine levels [53]. Thus, these features of MOCS2 are connected to the functions of xanthine dehydrogenase (XDH) via polyamine transfer (Figure 2).

XDH is a molybdenum enzyme involved in the terminal two reactions in the purine catabolic pathway: oxidation of hypoxanthine to xanthine and oxidation of xanthine to uric acid [58]. It was found that XDH is essential for mRNA loading of 539 genes through RNA sequencing of polysome fractions from XDH-knockdown cells [43]. Among these genes were found *RING1B* and *BMI1*, which encode subunits of polycomb repressive complex 1 (PRC1) (Almeida et al., 2017), and PCGF5, which encodes a subunit of noncanonical PRC1 complex (hereafter referred to as vPRC1). Importantly, translation of MOCS2 is also XDH-dependent [43]. MOCS2 and XDH mediate the association of PRC1/vPRC1 with Box C/D, which are small nucleolar ribonucleoproteins. Therefore, nucleotide metabolism regulated by MOCS2 and XDH is essential for mRNA loading of genes encoding subunits of PRC1/vPRC1 onto polysomes and influences RNA synthesis. It turns out that the metabolic pathways regulated by two molybdenum enzymes, SUOX and XDH, are connected by features of MPT synthase. XDH may be facilitated by MPTAC to accelerate the translation of certain genes when sulfide toxicity is elevated.

## 7. The Association of ATAC with MPTAC Is Lost in Neurodegenerative Diseases

ROS is generated by alkylating agents that modify N^7^-guanine (N^7^G) to N^7^-methylguanine (N^7^meG) and O^6^-guanine (O^6^G) to O^6^-methylguanine (O^6^meG) [59]. Suppression of ROS generation by MPTAC is part of the DNA alkylation damage response [60]. The heterodimer complex MutSα, consisting of MSH2 and MSH6, binds O^6^meG:C in mismatch repair (MMR) [61]. MutSα containing unphosphorylated MSH6 has higher affinity for O^6^meG-T than for G-T mismatches, inducing DNA damage signaling [59,61] (Figure 2). Importantly, unphosphorylated MSH6 is stabilized by its interactions with MPTAC and ATAC [60]. Translation of *HMGCS1*, which encodes the enzyme 3-hydroxyl-3-methylglutaryl-CoA synthase 1, regulates the mevalonate pathway upstream of sterol biosynthesis, requires acetyl-CoA, and is MPTAC-dependent [60]. The interactions between MutS, MPTAC, and ATAC, in turn, promote sterol biosynthesis, leading to an anti-inflammation response [60] (Figure 2).

Remarkably, these interactions are disrupted in Fragile X-associated disorders (FXDs). FXDs occur in individuals with a CGG repeat expansion in the 5′ untranslated region of *FMR1* [62], and they include Fragile X syndrome (FXS) (>200 CGG repeats, referred to as “full mutation”) [63], Fragile-X-associated tremor/ataxia syndrome (FXTAS) (55–200 CGG repeats, referred to as “premutation”), and Fragile-X-associated primary ovarian insufficiency (FXPOI) (premutation) [62]. FXDs are characterized by inherited intellectual disability or cognitive disfunction [63]. FXS is a specific genetic risk factor in autism spectrum disorder (ASD) [64]. It was found that MPTAC was disrupted in lymphoblastoid cell lines (LCLs) from some FXD patients [60]. In addition, the interaction between MSH6 and ATAC was found to be lost in LCLs from other FXD patients [60]. Therefore, some FXD patients may have alkylation damage resistance due to the impairment of MPTAC and ATAC. Notably, the activity of xanthine oxidase (XO), which is converted from XDH in the inflammatory environment [58] (Figure 2), is increased in LCLs not only from FXS patients, but also from non-FXS-diagnosed patients with FXS family members compared to LCLs from healthy donors [61]. Conversion of XDH into XO yields superoxide anions (O_2_^•–^) or hydrogen peroxide (H_2_O_2_) [58] (Figure 2), suggesting that alkylating agents are increased in these FXD patients. Therefore, an insight into the causes of elevated inflammatory response in FXD patients’ cells is destabilization of MutSα. Consistently, it has been shown that total cholesterol and low- and high-density lipoprotein are reduced in male FXS cohorts relative to the age-adjusted healthy population [65]. Immune-mediated diseases such as autoimmune thyroid disorder occur more frequently in females carrying an *FMR1* premutation than in age-matched female noncarriers [66]. An increase in Aβ deposition was observed in *Fmr1*-knockout mice [67]. Hence, malfunctions of MPTAC and ATAC may be involved in the clinical characteristics of FXD.

Overall, the functions of MPTAC associating with XDH appear to be double-faced. Increased XO activity, producing superoxide ions and hydrogen peroxide in an inflammatory environment, can induce apoptosis. However, XDH is also required for *MOCS2* mRNA loading onto polysomes, leading to promotion of MOCS2 protein expression, which is required for DNA alkylation damage signaling via MutSα. Therefore, XDH may function to remove toxic cells to stop the passage of mutations. XDH also contributes to the reduction of alkylating agents through MPTAC (Figure 2).

The expression levels of molybdenum cofactor sulfurase (MOCOS) were lower in the nasal olfactory stem cells of 11 adults with ASD than in 11 age- and gender-matched adult controls [68]. MOCOS sulfurates the Moco domains in XDH and aldehyde oxidase (AOX1) to activate these enzymes [69,70]. However, an association of alteration of XDH activities with ASD was not observed in this cohort study [68]. ASD diagnosis is determined based on major clinical characteristics, and FXD patients may exhibit characteristics that are distinct from those in ASD patients. Importantly, COSMOS, an antisense, long, noncoding RNA of MOCOS, plays a role in neuronal maturation [71]. In MoCD, rapidly progressing neurodegeneration leads to lethality in early childhood [72]. However, it is still unknown whether Moco biosynthetic enzymes directly regulate the functions of molecules that are responsible for neuronal development.

## 8. Prospects

Increases in the levels of inosine, hypoxanthine, and xanthine (metabolites in the purine salvage pathway) have been found in both MoCD [73] and Huntington’s disease (HD) [74]. HD is an inherited disease caused by the expansion of the CAG repeats that encode polyglutamine (polyQ) within *huntingtin* [75], and it is characterized by motor dysfunction and progressive dementia [74]. It remains unknown whether Moco biosynthetic enzymes are involved in late-onset neurodegenerative diseases, including HD. The progress of mild cases of MoCD has been shown to be suppressed by dietary restriction [76]. Therefore, it would be important to study features of Moco biosynthetic enzymes as metabolic regulators in late-onset neurodegenerative diseases. Further studies of Moco biosynthetic enzymes in terms of genetic factors (mutations) and DNA alkylation damage responses in nucleotide repeat and other neurodegenerative diseases will contribute to the understanding of the mechanisms underlying these diseases.

## Data Availability

Not applicable.
